# Expression Characteristics, Immune Signature, and Prognostic Value of the SOCS Family Identified by Multiomics Integrative Analysis in Liver Cancer

**DOI:** 10.1002/cnr2.2161

**Published:** 2024-09-22

**Authors:** Zhitao Dong, Binghua Dai, Rui Wu, Kunpeng Fang, Chengjun Sui, Li Geng, Jiamei Yang

**Affiliations:** ^1^ Department of Special Treatment Shanghai Eastern Hepatobiliary Surgery Hospital Shanghai China

**Keywords:** bioinformatics analysis, biomarker, liver cancer, prognosis, suppressor of cytokine signaling

## Abstract

**Background:**

Hepatocellular carcinoma (HCC) is a prevalent malignancy with a high mortality rate worldwide. Suppressor of cytokine signaling (SOCS) family members play important roles in the proliferation, metabolism, and immunity of HCC cells by regulating cytokines and growth factors. However, it remains uncertain whether the level of SOCS family members can affect the prognosis of HCC patients.

**Aims:**

This study aimed to comprehensively assess the role and mechanisms of SOCS family members in the development of HCC and to guide clinical selection.

**Methods:**

We investigated the expression levels of SOCS family genes in HCC patients and their associations with various clinicopathological characteristics. We also utilized a public database to analyze the changes in the expression, potential functions, transcription factors, and immune invasion of SOCS family members. Additionally, we examined the prognostic value of the SOC family for HCC and its correlation with the SOC family and ferroptosis‐related genes.

**Results:**

This study revealed that the expression of SOCS2‐7, and CISH was downregulated in HCC. The SOCS4, SOCS5, and SOCS7 genes were associated with the clinicopathological features of HCC patients. SOCS family genes are mainly related to the PIK3R3, GHR, and TNS4 pathways. Additionally, this study revealed that STAT3, PPAR‐gamma 2, and IRF‐2 are important transcription factors that regulate SOCS family members. The expression levels of SOCS family members are closely related to immune infiltration in liver cancer. The study also indicated that SOCS2 and SOCS4 are risk‐related genes for predicting the prognosis of patients with liver cancer. Finally, this study suggested that the SOCS2 gene may be involved in the development and progression of HCC.

**Conclusion:**

Our study enhances the current understanding of SOCS gene function in liver cancer and can help clinicians select appropriate drugs and predict the prognosis of HCC patients.

## 1 Introduction

Hepatocellular carcinoma (HCC) is one of the most common malignant tumors, accounting for 5% of all newly diagnosed cancers worldwide. It is estimated that HCC ranked sixth in incidence and third in death toll in 2020 [1]. Because of the high incidence and intractability of HCC at present, approximately 810 000–830 000, individuals worldwide die from liver cancer every year. Because HCC is insidious and has no symptoms in early pathology, most patients are diagnosed at a late stage, with high resistance to chemotherapy and a low success rate of radical surgery. Although the prognosis of HCC patients is poor, significant progress has been made in comprehensive and palliative treatment, including hepatectomy, local ablation, radiotherapy, chemotherapy, and liver transplantation.

Suppressor of cytokine signaling (SOCS) family members play important roles in liver cancer cell proliferation, metabolism, and immunity and have potential therapeutic effects in cancer treatment.

The SOCS family is a set of intracellular proteins that normally block the JAK/STAT pathway, which is involved in the downstream signaling of cytokines [2, 3]. Since their discovery in 1999, eight members of SOCS, including cytokine‐induced SH2 (CIS) and SOCS1‐7 [4], have been identified as major regulators of cytokines.

SOCS proteins contain the same structure as the C‐terminal domain (CTD), the center SH2, the SOCS box, and the sequence *N*‐terminal domain (NTD) [5]. In the SOCS family, SOCS‐BOX can form a stable complex with the elongated proteins B/C and assemble E3 ubiquitin ligase complexes for ubiquitination. There is evidence that SOCS family genes might be critical for the development of several human diseases, especially several malignant tumors [2].

Functionally, SOCS1‐3 and CISH were found to regulate the JAK/STAT pathway, which is a key factor in the occurrence and progression of some malignant cancers [6]. SOCS1 and SOCS3 were found to directly regulate the JAK kinase pathway. To date, there have been few studies on SOCS4‐7, and the specific functions of SOCS4‐7 are still unclear [7, 8, 9]. They may also play important roles by targeting multiple proteins. Zhang et al. reported that SOCS5 can promote metastasis by activating the PI3K/Akt/mTOR signaling pathway in liver cancer [10]. SOCS6 was found to inhibit the progression of lung cancer [11]. Several studies have revealed the relationships among several SOCS family members, the JAK/STAT pathway and ferroptosis‐related pathways in liver cancer [12]. However, the relationship between SOCS expression and prognosis in HCC patients is still unclear.

Recently, with the development of second‐generation prediction techniques, the role of SOCS family genes has been investigated [2, 13, 14]. In this study, we comprehensively explored the genomic, transcriptome, immune signature, clinical diagnostic, prognostic, and therapeutic value of the SOCS family in HCC. Our findings will enhance the current understanding of SOCS gene family function in liver cancer and will help clinicians select appropriate drugs and predict the prognosis of HCC patients.

## 2 Methods

In order to study the expression of SOCS family genes, the normalized RNAs seq data and related clinical data of LIHC samples (*n* = 374) and normal liver tissues (*n* = 50) are got from UCSC database [15] (http://xena.ucsc.edu/) of August 21, 2022. The messenger RNA (mRNA) expression data were obtained from UCSC in HTSeq FPKM format. The clinicopathological details of 374 LIHC patients were shown in Table 1.

**TABLE 1:** Clinicopathological details of 374 HCC patients.

Characteristic	Levels	Overall
*n*		374
T stage, *n* (%)	T1	183 (49.3%)
	T2	95 (25.6%)
	T3	80 (21.6%)
	T4	13 (3.5%)
N stage, *n* (%)	N0	254 (98.4%)
	N1	4 (1.6%)
M stage, *n* (%)	M0	268 (98.5%)
	M1	4 (1.5%)
Pathologic stage, *n* (%)	Stage I	173 (49.4%)
	Stage II	87 (24.9%)
	Stage III	85 (24.3%)
	Stage IV	5 (1.4%)
Tumor status, *n* (%)	Tumor free	202 (56.9%)
	With tumor	153 (43.1%)
Gender, *n* (%)	Female	121 (32.4%)
	Male	253 (67.6%)
Race, *n* (%)	Asian	160 (44.2%)
	Black or African American	17 (4.7%)
	White	185 (51.1%)
Age, *n* (%)	≤60	177 (47.5%)
	>60	196 (52.5%)
Weight, *n* (%)	≤70	184 (53.2%)
	>70	162 (46.8%)
Height, *n* (%)	<170	201 (58.9%)
	≥170	140 (41.1%)
BMI, *n* (%)	≤25	177 (52.5%)
	>25	160 (47.5%)
Residual tumor, *n* (%)	R0	327 (94.8%)
	R1	17 (4.9%)
	R2	1 (0.3%)
Histologic grade, *n* (%)	G1	55 (14.9%)
	G2	178 (48.2%)
	G3	124 (33.6%)
	G4	12 (3.3%)
Adjacent hepatic tissue inflammation, *n* (%)	None	118 (49.8%)
	Mild	101 (42.6%)
	Severe	18 (7.6%)
AFP (ng/mL), *n* (%)	≤400	215 (76.8%)
	>400	65 (23.2%)
Albumin (g/dL), *n* (%)	<3.5	69 (23%)
	≥3.5	231 (77%)
Age, median (IQR)		61 (52, 69)

We obtained RNA‐seq and clinical data of 374 LIHC and 50 normal liver tissues from The Cancer Genome Atlas (TCGA) database using the University of California Santa Cruz Xena platform. Protein–protein interaction network analysis was performed using STRING database version 11.0. Potential transcription factor targets were identified using GeneCards database version 5.0. Gene expression correlation analysis and pathway enrichment analysis were conducted using R software packages including “limma,” “clusterProfiler,” and “ggplot2.” Immune infiltration analysis was performed using TIMER database. Drug sensitivity analysis utilized data from Genomics of Drug Sensitivity in Cancer (GDSC) database.

### 2.1 Cell Culture and Treatments

We purchased the human LIHC cell line HepG2 from the Shanghai Institute of Cell Biology. HepG2 cell lines were cultured in RPMI‐1640 medium, which was supplemented with 10% fetal bovine serum (Gibco, United States) and 1% penicillin/streptomycin (Gibco, United States).HepG2 cell grew in a humid environment of 37°C and 5% CO_2_.

The human HCC cell line HepG2 used in this study was obtained from Shanghai Cell Bank. Short tandem repeat (STR) profiling was performed to authenticate the identity of HepG2 cells, and the results were consistent with records in the ATCC database. Additionally, HepG2 cells were routinely tested for mycoplasma contamination on a monthly basis using a mycoplasma PCR detection kit, with all results being negative.

### 2.2 Cell Transfection

In order to construct SOCS2 overexpression plasmid, we synthesized human SOCS2 complementary DNA and cloned it into pCDNA3.1 vector by BBI LIFE SCIENCE (Shanghai, China). Empty plasmids served as negative controls. According to manufacturer's instructions, HepG2 cell lines were transfected with jetPRIME (polyplus transfection, France).

### 2.3 Cell Proliferation, Migration, and Invasion Assays

Cell counting kit (CCK‐8, Biolite, USA) is used for cell viability detection. In short, cells are seeded in 96 well plates with a density of 3000 cells per well. We add 10‐mL of CCK‐8 reagent to each well at 0, 24, 48, 72, 96 h, incubate for 2 h, and calculate the absorbance at 450 nm with an automatic enzyme‐linked immunosorbent assay. The transwell chambers (8‐μm pore size, Corning) without Matrigel (Thermal, United States) or with Matrigel were used for cell migration assays or invasion assays, respectively. 4 × 104 HepG2 cells were placed in 200 μL RPMI‐1640 medium and placed in the top chamber. A total of 750 μL of RPMI‐1640 medium containing 20% fetal bovine serum was added into the lower cavity. The HepG2 cells were cultivated for 16 h (migration test) and 48 h (invasion test), and then HepG2 cells placed in the roof chamber were scraped with cotton swabs, and were photographed and counted with crystal violet staining. The CCK‐8 assay was performed in triplicate across three independent experiments. Data are presented as mean ± standard deviation (SD) and were analyzed using Student's *t*‐test for statistical significance.

### 2.4 Western Blot Analysis

The buffer was lysed by radioimmunoprecipitation (Sigma Aldrich; Merck). The protein concentration was calculated with BCA protein quantitative kit (Abcam, English). The total protein was then separated by 12% sodium dodecyl sulfate polyacrylamide gel electrophoresis and transferred to a polyvinylidene fluoride membrane (Millipore, Germany) where it was incubated with the first antibody overnight. The images were obtained through the FluorChem system. Antibodies used in this study included anti‐SOCS2 (1:2000, Proteintech), beta‐actin (1:2000, Proteintech), and horseradish peroxids‐mediated secondary goat anti‐mouse (1:5000, Biosharp).

### 2.5 Survival Analysis

In order to evaluate the overall survival (OS) of eight SOCS genes at different expression levels, we used “lima” and “survival” R packages for Kaplan–Meier analysis. To put it simply, we use the “limma” software package to determine the high and low expression groups of SOCS, and then integrate the clinical data with the RNA seq data downloaded from the UCSC database. A total of 374 LIHC patients were divided into different subgroups according to the different expression levels of SOCS gene expression. Finally, Kaplan–Meier analysis was used to evaluate OS of different subgroups. Multivariable Cox regression analysis was constructed to evaluate the prognostic value of SOCS family members in LIHC patients.

### 2.6 Statistical Analysis

In our study, we used a variety of statistical tests to validate our results. The Student's *t*‐test compared continuous variables, such as SOCS gene expression levels, between normal and cancerous liver tissues. Chi‐square or Fisher's exact tests were used for categorical data, such as tumor stages, depending on the sample size. The Wilcoxon rank sum test was applied when data was not normally distributed, particularly for comparing SOCS expression across age and gender. Survival analysis was performed using Kaplan–Meier curves and log‐rank tests, while Cox regression helped identify factors influencing HCC prognosis. All the data in this experiment were analyzed using IBM SPSS Statistics 22.0 and R software 4.1.3. *p* < 0.05 was considered statistically significant.

## 3 Results

### 3.1 Expression Analysis of SOCS Family Members in Hepatocellular Carcinoma and Normal Liver Tissues

**FIGURE 1:**
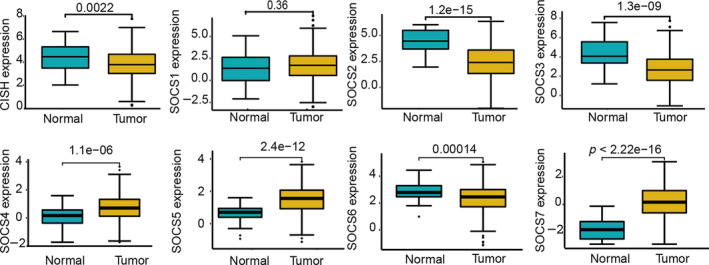
Expression levels of SOCS family members in LIHC and correlation with different clinicopathological features. Expression levels of SOCS family members in LIHC were determined by UCSC Xena database: compared to normal liver tissues, mRNA expression levels of SOCS2 (*p* = 1.2E‐15), SOCS3 (*p* = 1.3E‐09), SOCS6 (*p* = 0.00014), and CISH (*p* = 0.0011) decreased in HCC, while expression levels of SOCS4 (*p* = 1.1e‐06), SOCS5 (*p* = 2.4E‐12), and SOCS7 (*p* = 2.22E‐16) significantly increased.

The differences in the RNA expression levels of SOCS family members between normal liver tissues and HCC tissues were first examined. As shown in Figure 1, compared with those in normal liver tissues, the mRNA expression levels of SOCS2 (*p* = 1.2E‐15), SOCS3 (*p* = 1.3E‐09), SOCS6 (*p* = 0.00014) and CISH (0.0011) decreased in HCC tissues, while the expression levels of SOCS4 (*p* = 1.1e‐06), SOCS5 (*p* = 2.4E‐12), and SOCS7 (*p* = 2.22E‐16) increased significantly. Among the different age groups, SOCS5 expression decreased significantly in elderly patients (*p* = 0.015), while SOCS family member expression did not significantly differ among the different age groups (*p* > 0.05) (Supporting Information). Among the different sex groups, no significant difference was found in the expression of the CISH gene (*p* > 0.05) (Supporting Information). These experimental results suggested that SOCS family members may play a key role in tumorigenesis and may serve as biomarkers of prognosis in HCC patients.

### 3.2 Changes in Gene Expression Levels, Coexpression Analysis, Network Analysis of Protein–Protein Interactions and Methylation Levels of SOCS Family Genes in HCC Patients

**FIGURE 2:**
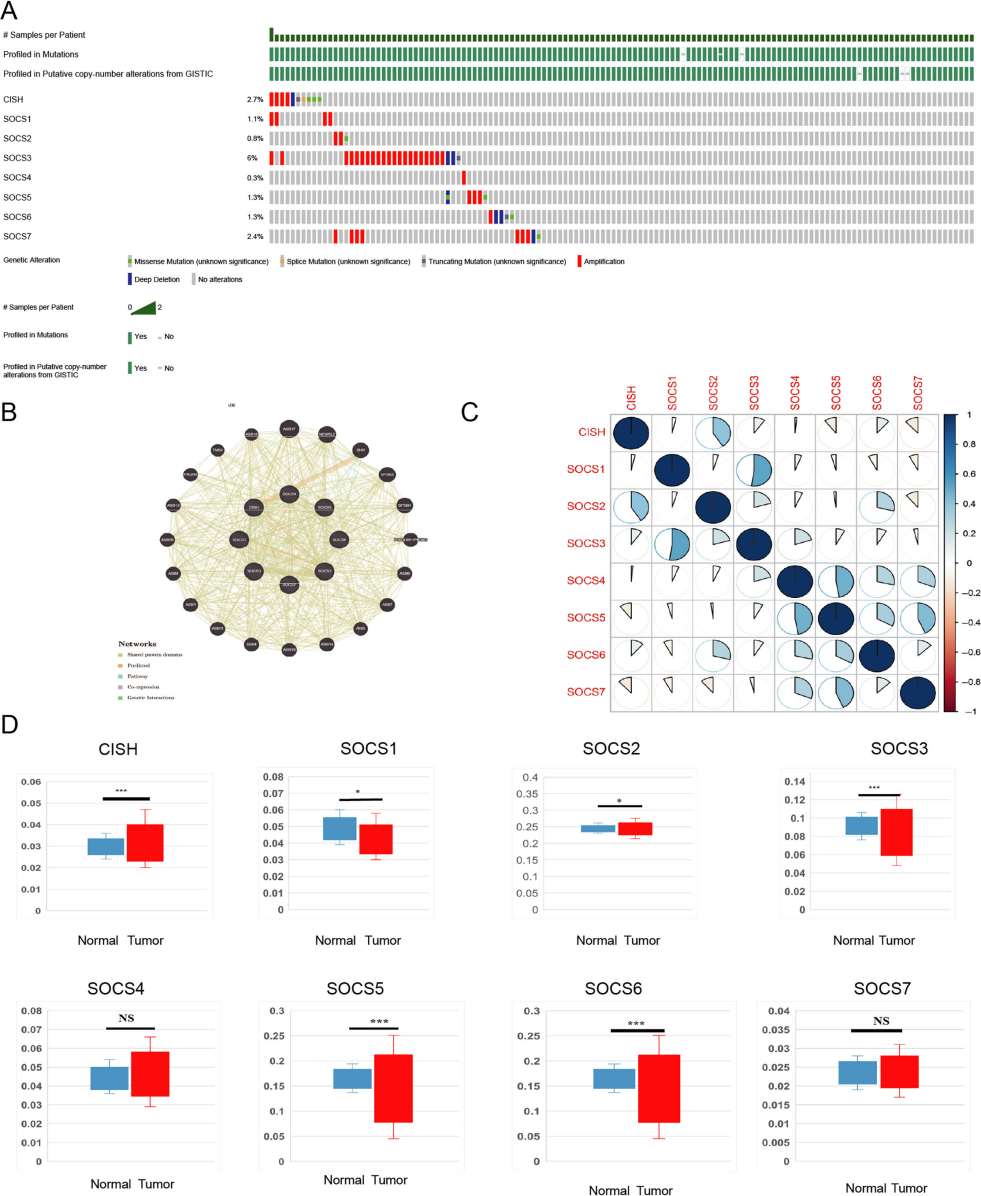
(A) Gene expression and mutation analysis of SOCS family members in LIHC: a gene expression and mutation analysis (cBioPortal). (B) The results of PPI network analysis suggested that these SOCS family members are mainly related to PIK3R3, GHR, TNS4, and TNS4 pathway. (C) Potential co‐expression analysis indicated a significant correlation was observed among SOCS family members. The bluer the color, the stronger their positive correlation and the redder the color, the stronger their negative correlation. (D) Methylation level of SOCS gene family promoters in HCC patients.

Changes in the expression of genes encoding different SOCS family members in HCC patients were evaluated by using the cBioPortal for Cancer Genomics. SOCS1 alterations through SOCS7 and CISH alterations were detected in liver cancer tissues at the following frequencies: SOCS1, 1.1%; SOCS2, 0.8%; SOCS3, 6%; SOCS4, 0.3%; SOCS5, 1.3%; SOCS6, 1.3%; SOCS7, 2.4%; and CISH, 2.7% (Figure 2A).

Next, we used the STRING website to construct a PPI network to evaluate the correlations between these SOCS family members and analyzed the potential functions and associated signaling pathways of eight SOCS family genes via the GeneMANIA website. These SOCS family members are mainly associated with the PIK3R3, GHR, and TNS4 pathways (Figure 2B).

Subsequently, we evaluated the possible relationships between the expressions of the eight SOCS family members (Figure 2C). The results showed that CISH was positively correlated with SOCS2, SOCS3, and SOCS6 but negatively correlated with SOCS5 and SOCS7; SOCS1 was positively correlated with SOCS3; SOCS2 was positively correlated with CISH, SOCS3, and SOCS6; SOCS3 was positively correlated with SOCS1, SOCS2, and SOCS4; SOCS4 was positively correlated with SOCS3, SOCS5, SOCS6, and SOCS7; SOCS5 was positively correlated with SOCS4, SOCS6, and SOCS7 but negatively correlated with SOCS1; SOCS6 was positively correlated with SOCS2, SOCS4, and SOCS5; SOCS7 was positively correlated with SOCS7, SOCS4, SOCS5, and SOCS6, while SOCS7 was negatively correlated with CISH and SOCS2.

Furthermore, the methylation levels of SOCS family gene promoters in HCC were analyzed via the cBioPortal Web Database (Figure 2D). The results indicated that the promoter methylation levels of SOCS1 (*p–* = 7.379E‐07), SOCS3 (*p* = 4.534E‐10), SOCS5 (*p* = 1E‐12), SOCS6 (*p* = 5.534E‐11), and CISH (2.273E‐05) were lower in cancer tissues than in normal tissues. These changes in promoter methylation could indicate possible differences in SOCS family gene expression levels in HCC patients.

### 3.3 Prediction of SOCS Family Member Transcription Factor Targets

The potential TF targets of SOCS family genes were explored using the GeneCards website. As shown in Table 2, the key transcription factors involved in SOCS1 may include AP‐1, ATF‐2, CREB, deltaCREB, and STAT3; the key transcription factors involved in SOCS2 may include GATA‐1, ITF‐2, PPAR‐gamma 1, PPAR‐gamma 2, STAT5B, and Tal‐1 beta; the key transcription factors involved in SOCS3 may include AP‐4, NRSF form 2, Pax‐5, c‐Rel, GATA‐3, c‐Myb, NRSF form 1, PPAR‐gamma 2, STAT5B, and PPAR‐gamma 1; the key transcription factors involved in SOCS4 may include AML1a, CREB Ik‐1, AREB6, POU3F2 (N‐Oct‐5a), POU3F2 (N‐Oct‐5b), STAT3, POU3F2, and SEF‐1 (1); the key transcription factors involved in SOCS5 may include NF‐AT3, NF‐AT4, NF‐AT, NF‐AT1, NF‐AT2, and PPAR‐gamma 2; the key transcription factors involved in SOCS6 may include EBPalpha C/EBPbeta, CUTL1, Evi‐1, IRF‐2, MIF‐1, POU3F2, POU6F1 (c2), and STAT3; the key transcription factors involved in SOCS7 may include GATA‐1, MAZR, POU2F1, ATF‐2, c‐Jun, c‐Myb, Egr‐2, STAT3, POU2F1a, and SEF‐1 (1); and the key transcription factors involved in CISH may include NRSF form 1, STAT3, STAT5A, STAT5B, GR, GR alpha, and GR beta.

**TABLE 2:** Transcription factor targets of SOCS family members.

SOCS family	Transcription factor targets
SOCS1	AP‐1, ATF‐2, CREB, deltaCREB, STAT3
SOCS2	GATA‐1, ITF‐2, PPAR‐gamma 1, PPAR‐gamma 2, STAT5B, Tal‐1 beta
SOCS3	AP‐4, c‐Myb, c‐Rel, GATA‐3, NRSF form 1, NRSF form 2, Pax‐5, PPAR‐gamma 1, PPAR‐gamma 2, STAT5B
SOCS4	AML1a, AREB6, CREB Ik‐1, POU3F2, POU3F2 (N‐Oct‐5a), POU3F2 (N‐Oct‐5b), SEF‐1 (1), STAT3
SOCS5	NF‐AT, NF‐AT1, NF‐AT2, NF‐AT3, NF‐AT4, PPAR‐gamma 2
SOCS6	EBPalpha C/EBPbeta, CUTL1, Evi‐1, IRF‐2, MIF‐1, POU3F2, POU6F1 (c2), STAT3
SOCS7	ATF‐2, c‐Jun, c‐Myb, Egr‐2, GATA‐1, MAZR, POU2F1, POU2F1a, SEF‐1 (1), STAT3
CISH	GR, GR alpha, GR beta, NRSF form 1, STAT3 STAT5A, STAT5B

### 3.4 Correlation of Immune Infiltration With SOCS Family Member Expression in HCC Patients

**FIGURE 3:**
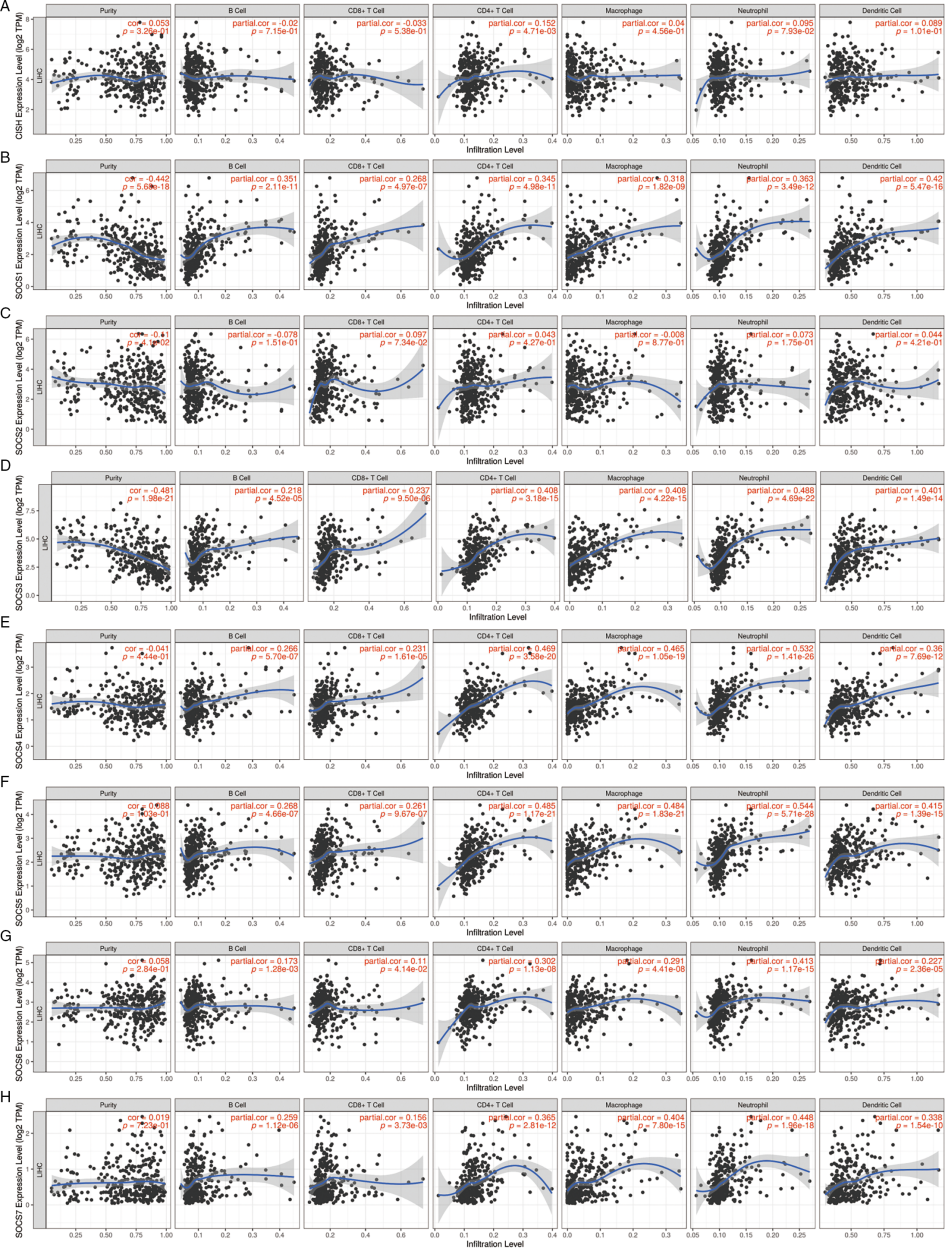
Correlation of SOCS expressions with immune infiltration level in LIHC. (A) CISH expression was positively associated with infiltration of CD8+ T cells and dendritic cells. (B) SOCS1 expression was positively associated with infiltration of dendritic and B cells. (C) SOCS2 expression was positively associated with infiltration of CD8+ T cells, CD4+ T cells, macrophages, and neutrophils and negatively associated with infiltration of B cells. (D) SOCS3 expression was positively associated with infiltration of CD4+ T cells, neutrophils, and dendritic cells. (E) SOCS4 expression was positively associated with infiltration of CD8+ T cells, CD4+ T cells, neutrophils, dendritic cells, and macrophages. (F) SOCS5 expression was positively associated with infiltration of all six types of immune cells. (G) SOCS6 expression was positively associated with infiltration of all six types of immune cells. (H) SOCS7 expression was positively associated with infiltration of B cells, CD4+ T cells, neutrophils, dendritic cells, and macrophages.

The correlation between immune infiltration and SOCS family member expression in HCC patients was assessed. Since previous studies have confirmed that members of the SOCS family are highly enriched in NK cell‐mediated cytotoxicity [16], we hypothesized that the SOCS family might participate in the tumorigenesis of HCC. Using the TIMER database, we further investigated the possible associations of SOCS proteins with immune infiltration (Figure 3). The gene level of CISH was significantly correlated with that of CD4+ T cells (*p* = 4.71e‐03).

### 3.5 Inhibitors of the Cytokine Signaling Family Have Been Implicated in the Prognosis of Liver Cancer Patients and Ferroptosis Gene Expression

**FIGURE 4:**
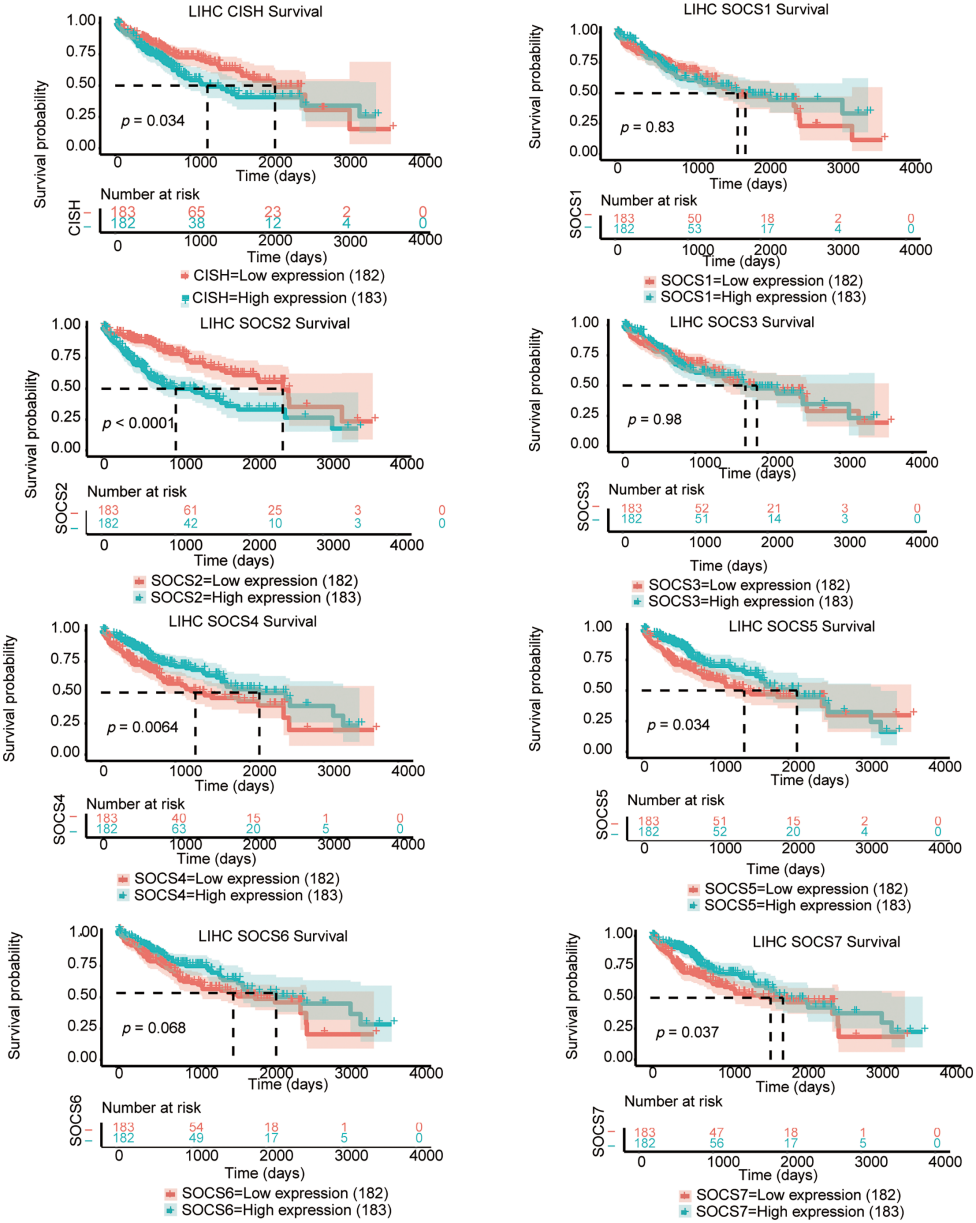
Survival analysis of SOCS family members in LIHC patients: prognostic impact on overall survival (OS). The expression levels of SOCS2 (*p* < 0.0001), SOCS4 (*p* = 0.0064), SOCS5 (*p* = 0.034), SOCS6 (*p* = 0.037), and CISH (*p* = 0.034) were found to be significantly associated with OS in HCC patients.

**FIGURE 5:**
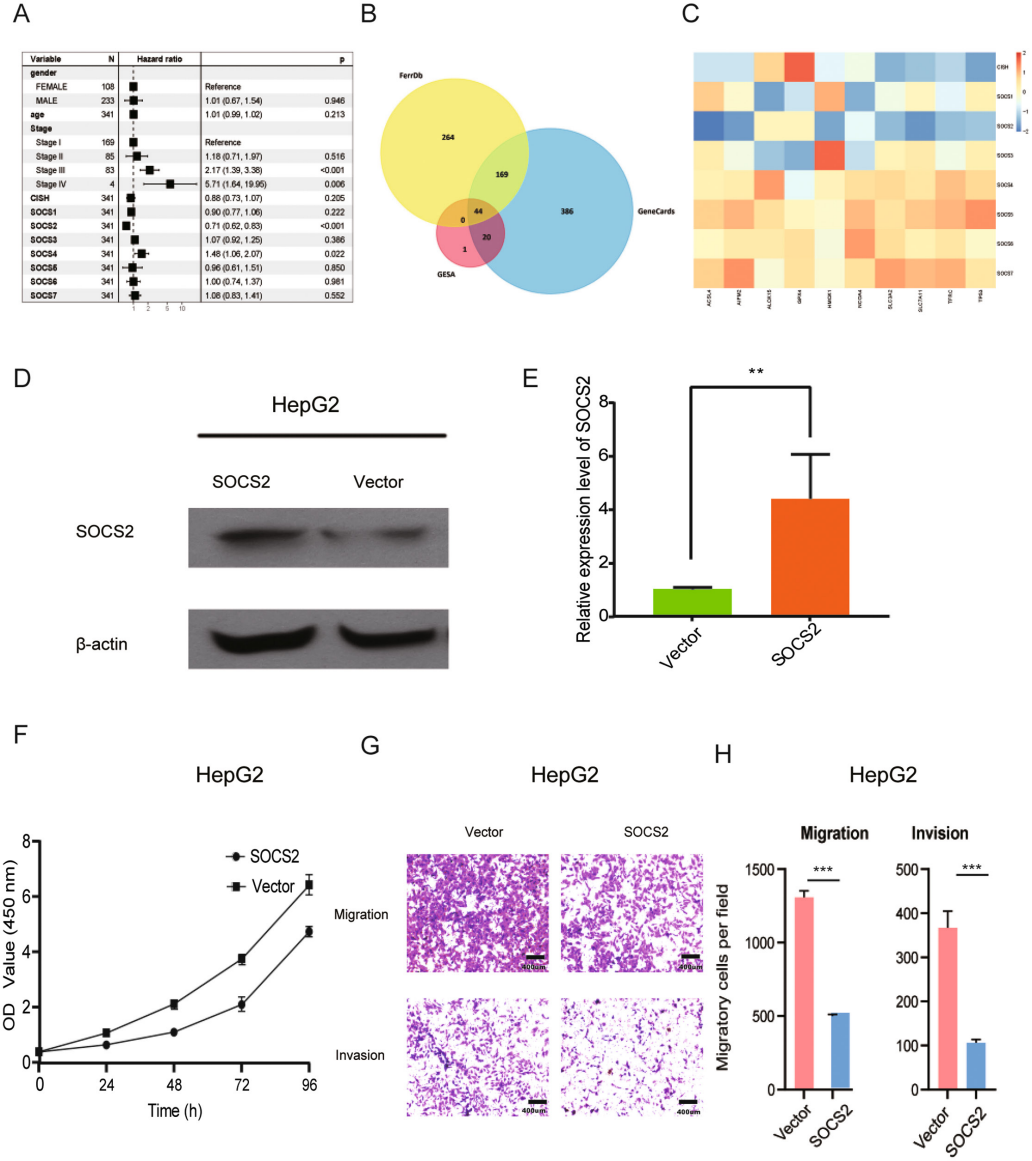
(A) Cox regression analysis indicated: SOCS2 and SOCS4 were associated with the OS of patients with LIHC. (B) Through VENN analysis, 64 related genes were found related to ferroptosis. (C) The GSEA analysis showed that SOCS family members is closely related to ferroptosis genes. (D) Western blot revealed that SOCS2 overexpression plasmids lead to an upregulation of SOCS2. (E) Analysis of the PCR revealed that the expression of SOCS2 was significantly upregulated in the SOCS2 overexpression plasmids. (F) SOCS2 inhibited cell proliferation by CCK‐8 assay. (G) Over‐ expression of SOCS2 inhibited migration and invasion in vitro.(H) The migratory cell per field in the SOCS2 overexpression group is less than that in the vector group (498 ± 21 vs. 1380 ± 65, *p* < 0.05). The invasive cell per field in the SOCS2 over‐expression group is less than that in the vector group (98 ± 8 vs. 373 ± 41, *p* < 0.05). Data are presented as mean ± SEM (*n* = 4). **p < 0.01, ***p < 0.001. Scale bar, 400 um.

The ability of eight SOCS family members to predict the prognosis of HCC patients was also assessed, and the results indicated that the levels of SOCS2 (*p* < 0.0001), SOCS4 (*p* = 0.0064), SOCS5 (*p* = 0.034), SOCS6 (*p* = 0.037), and CISH (p = 0.034) were significantly related to OS in HCC patients (*p* = 0.034) (Figure 4). Subsequently, we performed univariate and multivariate Cox regression analyses to validate the prognostic value of SOCS family genes. These results suggest that among the eight SOCS family genes, SOCS2 and SOCS4 are related to OS in patients with HCC (Figure 5A).

Then, we screened ferroptosis‐related genes through the FerrDb, GESA, and GeneCards databases. Through Venn analysis, we identified 64 related genes (Figure 5B). We analyzed the correlations between SOCS family genes and these genes. The results suggested that SOCS family members are closely related to ferroptosis genes (Figure 5C).

### 3.6 Cell Proliferation, Migration, and Invasion Are Inhibited When SOCS2 Is Overexpressed in Vitro

To further investigate the effect of SOCS2 on HCC, SOCS2 was transfected into HepG2 cells. The western blot results showed that SOCS2 overexpression led to significant upregulation of SOCS2 (Figure 5D). PCR analysis revealed that SOCS2 expression was significantly increased in the SOCS2‐overexpressing cells (Figure 5E). CCK‐8 experiments showed that SOCS2 overexpression inhibited cell proliferation (Figure 5F). Transwell assays showed that SOCS2 overexpression inhibited the migration and invasion of HepG2 cells (Figure 5G). The migratory cell per field in the SOCS2 overexpression group is less than that in the vector group (498 ± 21 vs. 1380 ± 65, *p* < 0.05). And also, the invasive cell per field in the SOCS2 overexpression group is less than that in the vector group (98 ± 8 vs. 373 ± 41, *p* < 0.05).

### 3.7 Potential Functional Analysis of SOCS Family Genes in Patients With Hepatocellular Carcinoma

**FIGURE 6:**
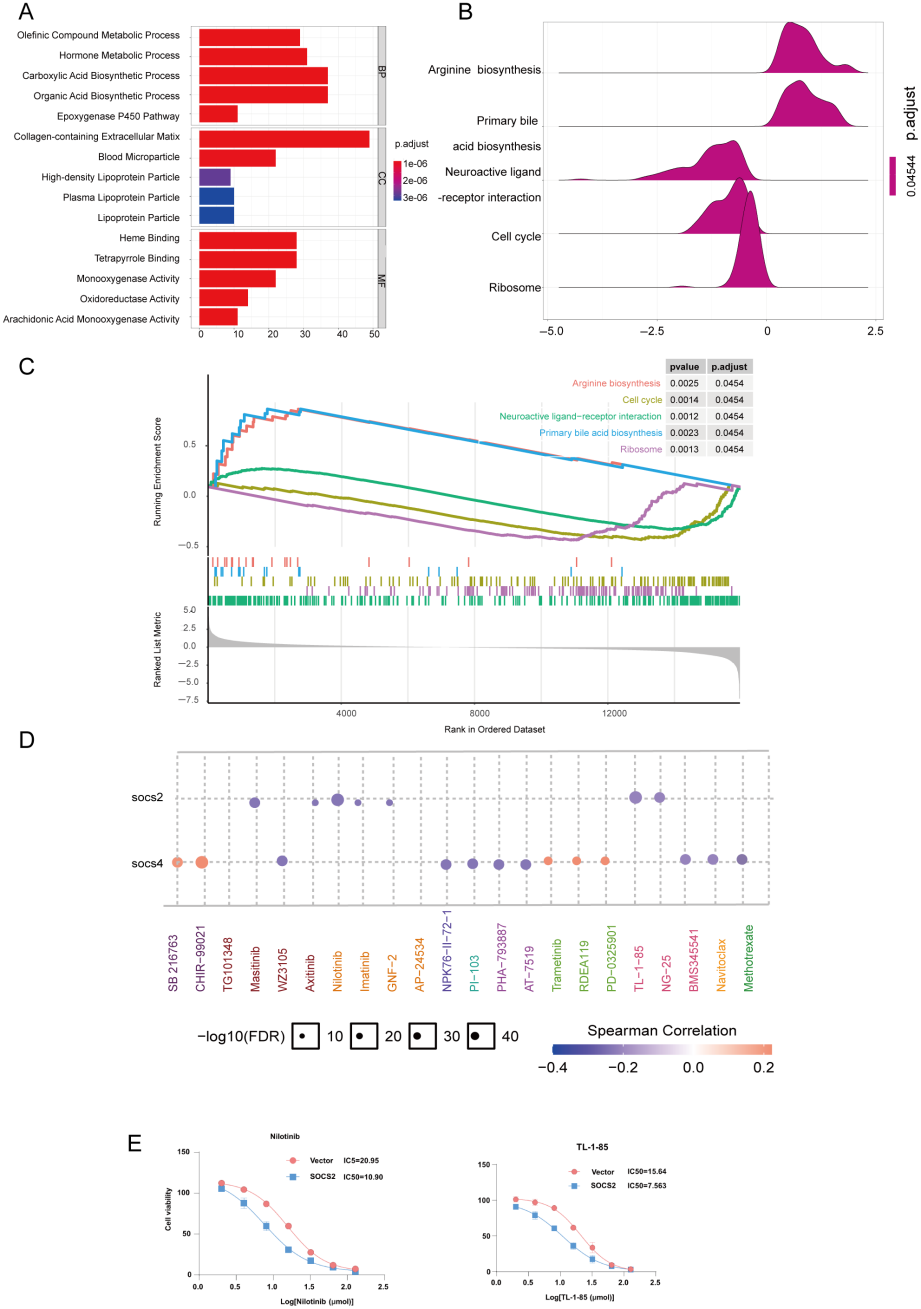
(A) Gene ontology (GO) enrichment analysis of SOCS2 and associated genes in hepatocellular carcinoma (HCC). The analysis highlights significant enrichment in biosynthetic processes (BP) such as olefinic and hormone metabolic processes, cellular components (CC) including blood microparticles and lipoprotein particles, and molecular functions (MF) like heme and tetrapyrrole binding activities. (B and C) Kyoto Encyclopedia of Genes and Genomes (KEGG) pathway analysis illustrating the involvement of SOCS2 in key tumor‐related pathways. Pathways include arginine biosynthesis, cell cycle regulation, and neural ligand‐receptor interaction, indicating potential roles in HCC pathogenesis and progression. (D) Drug sensitivity analysis correlating SOCS2 expression with response to therapy in HCC, based on IC50 data from the Genomics of Drug Sensitivity in Cancer (GDSC) database. High SOCS2 expression levels are associated with resistance to therapies such as masitinib and nilotinib, highlighting its potential as a biomarker for treatment stratification. (E) In nilotinib group, the IC50 concentration of SOCS2 was 10.90, significantly lower than the IC50 concentration of the control group (*p* < 0.05). In TL‐1‐85group, the IC50 concentration of SOCS2 was 7.563, significantly lower than the IC50 concentration of the control group (*p* < 0.05).

GO and KEGG enrichment analyses were subsequently conducted to further evaluate the potential functions of SOCS2 and related genes. Figure 6A shows that in the biosynthetic process (BP) category, SOCS2 and related genes were highly enriched in the olefinic compound metabolic process, hormone metabolic process, carboxylic acid biosynthetic process, organic acid biosynthetic process, and epoxygenase P450 pathway; in the cellular composition (CC) category, SOCS2‐related genes were highly enriched in blood microparticles, high‐density lipoprotein particles, collagen‐containing extracellular matrix, lipoprotein particles, and plasma lipoprotein particles; and in the molecular function (MF) category, SOCS2 and related genes were highly enriched in genes such as heme binding, tetrapyrrole binding, monooxygenase activity, oxidoreductase activity, and arachidonic acid monooxygenase activity. Additionally, KEGG pathway results revealed that SOCS2 was implicated in numerous classical tumor‐related pathways, including arginine biosynthesis, the cell cycle, neural ligand receptor interaction, primary bile acid biosynthesis, and ribosomes (Figure 6B,C).

Finally, drug sensitivity related to SOCS2 expression was analyzed via the GDSC database. As shown in Figure 6D, HCC patients with high levels of SOCS2 were resistant to combination therapy or small molecules such as nilotinib, TL‐1‐85, NG‐25, masitinib, axitinib, imatinib, and GNF‐2. We validated the drug sensitivity results of SOCS2 in vitro with nilotinib and TL‐1‐85 in Figure 6E. In nilotinib group, the IC50 concentration of SOCS2 was 10.90, significantly lower than the IC50 concentration of the control group (*p* < 0.05). In TL‐1‐85group, the IC50 concentration of SOCS2 was 7.563, significantly lower than the IC50 concentration of the control group (*p* < 0.05).The results indicated that the expression of SOCS2 was negatively correlated with the drug concentrations of nilotinib and TL‐1‐85.

## 4 Discussion

The SOCS family plays an important role in the regulation of cellular responses to cytokines and growth factors and is a major negative regulator of numerous signaling pathways, including the JAK/STAT pathway [3, 17, 18]. An increasing number of studies have suggested that SOCS family genes are key factors in the occurrence, development, invasion, and angiogenesis of malignant tumors [2, 19]. However, only a limited number of studies have shown that SOCS family members are associated with HCC [20], and the exact biological function of the SOCS family in HCC remains unclear.

First, we found that SOCS2‐SOCS7 and CISH were significantly elevated in HCC, and SOCS4, SOCS5, and SOCS7 were significantly increased with the progression of HCC. Similarly, SOCS proteins were reported to be responsible for negative feedback regulation of cytokine signaling and closely related to the development and liver regeneration of HCC. The decreased expression of SOCS1 and SOCS3 is associated with the late stage and poor prognosis of HCC [21]. And also, SOCS family members have also been found to play a role in breast cancer. Research shows that the expression levels of SOCS1, SOCS2, and SOCS3 are different in breast cancer tissue, which may be related to the heterogeneity of breast cancer tissue. SOCS3 may be a factor in the prognosis of breast cancer patients, while SOCS2 may be a potential therapeutic target [13].

Then, we evaluated the genetic changes in SOCS family genes in HCC. Elevated or reduced mRNA levels are the most frequent changes in SOCS family genes. These SOCS family members are significantly correlated with one another and these genes are mainly related to the JAK/STAT cascade, SH3/SH2 junction activity and signal junction activity [22]. Gene Ontology (GO) and Kyoto Encyclopedia of Genes and Genomes (KEGG) pathway enrichment analyses were carried out to further investigate the functions of SOCS family members. The results indicated that the SOCS and associated genes were enriched in the PIK3R3, GHR, TNS4, and TNS4 pathways. Khan et al. highlighted the importance of SOCS1 and SOCS3 as tumor suppressors in HCC. They found that the expression of these proteins is suppressed, potentially leading to the activation of oncogenic signaling pathways. By analyzing cancer genome atlas datasets, a study revealed a correlation between the expression of SOCS1 and SOCS3 and the prognosis of HCC patients, where high expression of SOCS1 was associated with a favorable prognosis [21].

Previous studies have shown that the PIK3R3/STAT3 pathway may play an important role in the regulation of embryonic development, hematopoiesis, the inflammatory response, and stem cell maintenance [18]. The abnormally activated PIK3R3/STAT3 signaling pathway is critical for many cancers [17, 18]. The SOCS family of genes, which are important regulators of PIK3R3/STAT3 signaling, can be used to develop molecularly targeted drugs to inhibit PIK3R3/STAT3 signaling in a variety of cancers with unique modes of action [23]. Our data indicated that the promoter methylation levels of SOCS1, SOCS3, SOCS5, SOCS6, and CISH were greater in normal tissues than in HCC tissues. Next, we analyzed the possible TFs that might be involved in the SOCS family. Our study revealed that STAT3, PPAR‐gamma 2, and IRF‐2 may regulate these SOCS genes as important transcription factors.

The correlation of SOCS genes with immunological infiltration was evaluated. The results showed that the immune infiltration of CD4+ T cells, CD8+ T cells, B cells, neutrophils, macrophages, and dendritic cells was closely related to SOCS family genes. Next, we assessed the prognostic value of SOCS family members in HCC, and the results indicated that SOCS2 and SOCS4 are risk‐related genes in patients with liver cancer. These results indicate that the SOCS family might be involved in the development of hepatic carcinoma. Meanwhile, studies have found that SOCS1‐3 and CISH regulate immune related signals, affecting lymphocyte polarization and activation of myeloid cells, which are particularly important in the tumor microenvironment. Disturbance of SOCS proteins may lead to inflammation and autoimmune diseases, thereby promoting the development of malignant tumors [20]. To reinforce this linkage, we plan to undertake a series of targeted experiments. These are designed to yield empirical evidence that will corroborate our preliminary findings, deepen our understanding of the immunological dynamics within HCC, and may pave the way for the formulation of future therapeutic strategies.

Abnormal expression of SOCS2 may be associated with the development, metastasis, and prognosis of HCC. We confirmed the antitumor effect of SOCS2 in vitro. The proliferation, migration, and invasion of cells were inhibited by SOCS2 overexpression. Our clinical data suggest that SOCS2, a key negative regulator of JAK/STAT signaling, may be associated with the risk of liver cancer progression. SOCS2 may be involved in the apoptosis or necrosis of cancer cells, which may eventually influence the progression of HCC [24]. However, the detailed function of SOCS2 in the regulation of cell death (RCD) requires further research.

Chen et al. reported that SOCS2 was significantly reduced in hepatic carcinoma tissues and that the expression of SOCS2 was associated with introhepatic metastasis and histology [25]. Ren et al. reported that SOCS2 is a potential target gene of miR‐196a and that miR‐196b and SOCS2 can enhance the expression of miR‐196 and miR‐96b in hepatic carcinoma [24]. Based on The Cancer Genome Map Project (TCGA), Li et al. developed a prognostic feature, and the results showed that the levels of SOCS2, β‐ureidopropionase (UPB1), and reticuloglobin 3 (RTN3) are independent predictors of HCC [26]. Compared with other combinations, low expression of SOCS2 and high expression of RTN3 were related to poorer survival. They also assessed the protein level in hepatic carcinoma and found that SOCS2 was decreased in patients with hepatic carcinoma.

Ferroptosis is a new type of RCD [27] that differs from apoptosis, necrosis, and cytophagy in morphology, genetics, and biochemistry. Ferroptosis is characterized by the accumulation of cytotoxic lipid peroxides [28]. Recently, inducing or regulating the expression of proteins associated with iron has become a promising method for preventing cancer cell death. GPX4, P53, and SLC7A11 are key factors in liver cancer [29]. Chen et al. reported that SOCS2 can enhance ferroptosis and radiosensitivity in HCC by promoting the ubiquitination of SLC7A11. These findings suggest that high SOCS2 expression is correlated with a better prognosis for HCC patients and that by promoting K48‐linked polyubiquitination degradation of SLC7A11, SOCS2 can serve as a potential target for HCC treatment [30].

However, it is not yet clear whether other potential proteins can regulate ferroptosis and thus influence the prognosis of hepatic carcinoma patients. In this study, we found that there was a close relationship between SOCS2 and ferroptosis‐associated genes, suggesting that SOCS2 might interact with ferroptosis. Our results indicate that SOCS2 is essential for the development of HCC. Saint Germain et al. reported that SOCS1 regulates senescence and ferroptosis by modulating the expression of p53 target genes [31]. These findings suggest that SOCS family members may be promising target genes for the treatment of liver cancer.

This research has several limitations. In this study, we speculated that SOCS2 may play multiple roles in the progression of hepatic carcinoma and may act as a suppressor gene. However, the underlying mechanism of SOCS2 and its exact role in HCC formation are unclear.

Considering the role of SOCS2 expression in modulating the response to pharmacological interventions in HCC, our analysis of the GDSC database has provided substantial evidence. As demonstrated in Figure 6, a distinct pattern of resistance to various therapies was evident among patients with increased SOCS2 expression. This resistance encompasses not only combination therapy but also a range of small molecule inhibitors, including masitinib, nilotinib, TL‐1‐85, NG‐25, axitinib, imatinib, and GNF‐2. The resistance linked to SOCS2 may reflect its regulatory influence on cellular pathways that contribute to drug resistance [32]. Alternatively, it could indicate a more aggressive tumor phenotype with intrinsic resistance to these treatments [33]. Investigating the underlying mechanisms by which SOCS2 expression affects drug sensitivity is crucial, as it may reveal key insights that could guide the development of more effective therapeutic strategies for HCC patients.

In summary, this study comprehensively evaluated the expression patterns, functional roles, and prognostic significance of SOCS family members in HCC through integrated multiomics analysis. Additionally, we explored the associations between SOCS family members and the clinical features and prognosis of patients with liver cancer using multidimensional approaches. The results showed that SOCS2 and SOCS4 were correlated with patient outcomes. Future research needs to fully elucidate the complex mechanisms by which SOCS2 and SOCS4 regulate the occurrence and progression of HCC. Taken together, our findings may facilitate the development of SOCS family proteins as prognostic biomarkers or therapeutic targets for liver cancer.

## Author Contributions

**Zhitao Dong:** writing – original draft (equal), data curation (equal), formal analysis (equal). **Binghua Dai:** data curation (equal), formal analysis (equal), resources (equal), software (equal). **Rui** **Wu:** methodology (equal), project administration (equal), validation (equal), writing – original draft (equal). **Kunpeng Fang:** investigation (equal), methodology (equal), software (equal). **Chengjun Sui:** formal analysis (equal), methodology (equal), supervision (equal). **Li Geng:** formal analysis (equal), methodology (equal), visualization (equal), writing – review and editing (equal). **Jiamei Yang:** project administration (equal), supervision (equal), writing – review and editing (equal).

## Conflicts of Interest

The authors declare no conflicts of interest.

## Supporting information


**Data S1.** Expression levels of SOCS family members in LIHC and correlation with different clinical features. (A) Correlation between expression levels of SOCS family members and age: SOCS5 expression significantly decreased in elderly patients (*p* = 0.015), while no significant differences were observed for other SOCS family members across different age groups (*p* > 0.05). (B) Correlation between expression levels of SOCS family members and sex: no significant differences in SOCS gene expression were found between different gender groups (*p* > 0.05).

## Data Availability

The data that support the findings of this study are available from the corresponding author upon reasonable request.
