# Supplementary figures and images for: Expression Characteristics, Immune Signature, and Prognostic Value of the SOCS Family Identified by Multiomics Integrative Analysis in Liver Cancer
Source: Cancer Rep (Hoboken). 2024 Sep 22;7(9):e2161. doi: 10.1002/cnr2.2161 (PMC11416904; doi:10.1002/cnr2.2161)

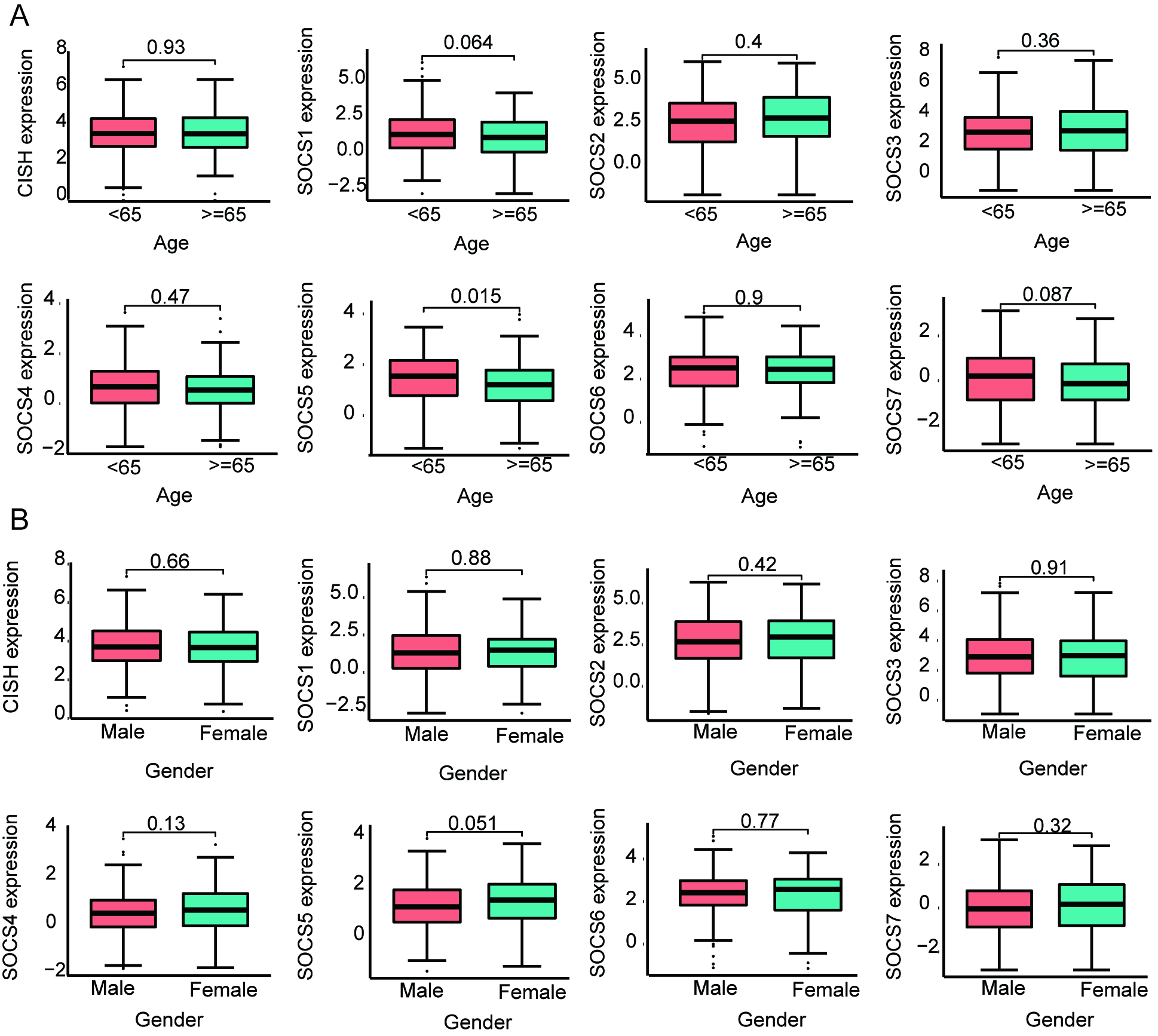

Supplement: Supplementary file 1 — Data S1. Expression levels of SOCS family members in LIHC and correlation with different clinical features. (A) Correlation between expression levels of SOCS family members and age: SOCS5 expression significantly decreased in elderly patients (p = 0.015), while no significant differences were observed for other SOCS family members across different age groups (p > 0.05). (B) Correlation between expression levels of SOCS family members and sex: no significant differences in SOCS gene expression were found between different gender groups (p > 0.05). [file CNR2-7-e2161-s001.tif]
